# Profiles of Quality of Life in a Homeless Population

**DOI:** 10.3389/fpsyt.2019.00010

**Published:** 2019-01-30

**Authors:** Lia Gentil, Guy Grenier, Jean-Marie Bamvita, Henri Dorvil, Marie-Josée Fleury

**Affiliations:** ^1^Department of Psychiatry, McGill University, Montreal, QC, Canada; ^2^Research Center, Douglas Mental Health University Institute, Montreal, QC, Canada; ^3^School of Social Work, Université du Québec à Montréal, Montreal, QC, Canada

**Keywords:** quality of life, homeless, cluster analysis, mental health disorders, substance use disorders, type of accommodation, health care service use variables

## Abstract

Quality of life (QOL) is a key indicator in mental health planning, program evaluation, and evaluation of patient outcomes. Yet few studies have focused on QOL in homeless populations. More specifically, research has yet to identify profiles of homeless individuals based on their QOL using cluster analysis. This study developed a typology of QOL for a sample of 455 homeless individuals recruited from 27 community and public organizations in Quebec (Canada). The typology was developed based on QOL scores, as well as sociodemographic, clinical, and service use variables. Study participants had to be at least 18 years old, with current or previous experience of homelessness. A questionnaire including socio-demographics, residential history, service utilization, and health-related variables was administered. Four clusters were identified using a two-step cluster analysis. QOL was highest in the cluster consisting of older women with low functional disability, and relatively few episodes of homelessness. The second cluster with high QOL scores included individuals living in temporary housing with relatively few mental health or substance use disorders (SUDs). The third cluster with low QOL included middle-aged women living in temporary housing, with criminal records, personality disorders, and SUDs. QOL was also lower in the fourth cluster composed of individuals with multiple homeless episodes and complex health problems as well as high overall service use. Findings reinforced the importance of disseminating specific programs adapted to the diverse profiles of homeless individuals, with a view toward increasing their QOL.

## Introduction

Quality of life (QOL) is one of the most important indicators in mental health planning, program evaluation, and assessment of patient outcomes (1). QOL is a heterogeneous concept that encompasses many areas of objective and subjective well-being (2). Objective QOL includes aspects of the physical environment and social functioning (2), whereas subjective QOL (SQOL) relates more to individual preferences, opinions, and life satisfaction (3). Research on QOL has been conducted with patients affected by mental health disorders (MHDs) (4–7); while studies on QOL and homelessness have focused on veterans (8–10), newly housed individuals who were previously homeless (11, 12), or homeless individuals with MHDs or substance use disorders (SUDs) (13, 14). However, few studies have focused on QOL in homeless populations.

QOL in homelessness may be linked to multiple variables including sociodemographic characteristics, clinical, and health care service use variables. Some studies have reported higher QOL among older homeless women (12, 13, 15); whereas having a criminal record has been found to negatively affect QOL (16). Compared with individuals who have experienced multiple episodes of homelessness, those experiencing a first homeless episode had higher QOL (12). Fewer days of homelessness were also associated with higher QOL (17). Individuals living in permanent supported housing such as Housing First (HF) programs, which integrate financial subsidies, case management, and a harm reduction philosophy tailored to individual needs, had higher levels of QOL compared with control groups consisting of emergency shelter users, or those residing in temporary housing or various forms of independent housing viewed as inadequate (18–20). In terms of clinical variables, homeless individuals with common MHDs (e.g., depression), serious MHDs (e.g., psychosis), SUDs, and personality disorders reported low QOL scores (13, 17, 21), similar to results for homeless individuals with high functional disabilities (22). While suicidal behaviors associated with MHDs and SUDs (23–25), and physical illnesses were also found to be prevalent in homeless populations (e.g., diabetes, cardiovascular disease) (26, 27), research has yet to investigate possible associations between these conditions and QOL. Regarding service use, one study found that being enrolled in outpatient services produced improved QOL scores in a homeless sample (28), while another identified improved QOL after enrolment in medical, employment or public support programs (17, 19). While homeless individuals with MHDs, SUDs or physical illnesses have tended to be high emergency room (ER) users, defined as four or more ER visits in a single year (29, 30) no study has identified associations between high ER use and QOL.

Cluster analysis is a useful method for establishing typologies (31), and may be used to investigate QOL among individuals experiencing homelessness. General profiles have been developed using cluster analysis among homeless individuals with both common and serious MHDs (32–34), co-occurring MHDs/SUDs (35), or physical illnesses (36), as well as among patients with serious MHDs using psychiatric services (33) and those using shelters over a multiple-year period (37–41). However, no known study has used cluster analysis to identify profiles of homeless individuals based on their QOL. Moreover, few cluster analyses in homelessness have taken into account the possible effects of sociodemographic characteristics such as criminal record, episodes of homelessness, and different types of accommodation on QOL. As well, clinical variables such as suicidal ideation or functional disability, and overall service use have hardly been considered in typologies of homelessness. The availability of a QOL typology based on sociodemographic, clinical, and service use variables for homeless populations would provide critical information and a deeper understanding that could inform the development of housing policies and services that capture the unique characteristics and needs of each group. Accordingly, the objective of this study was to build a typology based on QOL, sociodemographic and clinical variables, and service use for a sample of 455 homeless individuals in Quebec who were living in different types of accommodation.

## Materials and Methods

### Study Setting and Data Collection

The study was conducted in the two major urban areas of Quebec: Montreal and Quebec City. Montreal had the largest homeless population (*N* = 3,016), as well as 2,017 available beds (71% of the provincial total) in emergency shelters and transitional housing, while Quebec City had 262 beds (15%) (42). Recruitment was conducted in 27 public organizations, mainly community organizations (22 from Montreal and five from Quebec City). Twenty of these organizations offered housing resources; five with emergency shelters (29 beds per organization, on average); 12 with temporary housing resources (average 20 beds per organization), and three with permanent supported housing (total: 173 beds). The seven organizations without a housing component provided other essential services including food banks, day centers, leisure activities; employment or housing services, and financial or material support.

Eligibility requirements for participation in the study included current or previous experience of homelessness and age requirements (18 or older). No interested participant was excluded from the study, if eligible; but interviews sometimes had to be delayed for participants who were intoxicated or otherwise indisposed at recruitment time. Posters were displayed in common areas of the selected organizations. The project coordinator also recruited participants directly while present in sites where homeless people congregate, such as nearby cafés. Finally, researchers held information meetings with housing staff and enlisted their help with recruitment. There were four housing conditions: emergency shelters (overnight accommodation), temporary housing (3–12 month residency), and permanent housing (1 year to indefinite stay), either with financial and case manager support (Housing First), or without this support. Homeless individuals invited to participate in the study included 46 users of emergency shelters, 243 residents in temporary housing, 156 residents in permanent housing with support, and 52 permanent housing residents without support, for a total of 497 invited study participants.

Data were collected between January and September 2017 by trained interviewers. Interviews were conducted in the selected organizations, at participant apartments, or in quiet corners of local cafés or fast foods restaurants. Interviews usually took place on the same day, or day following initial contact, and averaged 90 min in duration. All study participants signed a consent form before undergoing an interview, and were told that their responses would remain confidential. The research ethics board of the Douglas Mental Health University Institute approved the multisite study protocol.

### Variables and Instruments

The questionnaire included socio-demographic information, as well as questions on residential history, service utilization, and clinical variables. The dependent variable, QOL, was measured using the Satisfaction with Life Domains Scale (SLDS), a frequently used instrument for QOL assessments in various fields. The SLDS was published initially by Baker and Intagliata in 1982 (43), and a French translation developed and validated (Cronbach Alpha = 0.92) by Caron (44). The SLDS assesses 20 domains of life satisfaction. As well, five stylized faces were used to represent a range of emotional states, from the saddest face (score = 1), to the happiest face (score = 5). Study participants were directed to select the face that corresponded to their current emotional state (43).

Table 1 presents all variables included in the study and the instruments used. Variables were based on the literature, and included socio-demographics: age, sex, criminal record, homelessness episodes, and type of accommodation; clinical variables: MHDs, SUDs, number of physical illnesses, suicidal ideation, and functional disability; and service use variables: has a family physician and frequency of the following services: community services (e.g., soup kitchen, day center, employment support program, support group, women's center); public services (local community service center; addiction rehabilitation center, hospital or other); and emergency room (ER).

**Table 1:** Variables and instruments.

**Variables**	**Instruments and references**	**Description**	**Number of items**	**Scoring/Range**	**Psychometric properties**
**VARIABLE OF INTEREST**					
Quality of life (QOL)	Satisfaction with Life Domains Scale (43, 44)	Note 1	20	Five-point Likert-scale; Range: 0–100	Cronbach's alpha = .0.92
**SOCIODEMOGRAPHIC VARIABLES**					
Age	Canadian Community Health Survey (CCHS) (45)	Note 2		Numerical	
Sex	CCHS (45)	Note 3		1 = male; 2 = female	
Criminal record	CCHS (45)	Note 4		Yes/No	
Homeless episodes	CCHS (45)	Note 5		Numerical	
Types of accommodation	CCHS (45)	Note 6		Emergency shelter Temporary housing Permanent housing with or without support	
**CLINICAL VARIABLES**					
Mental health disorders (MHDs)	M.I.N.I International Neuropsychiatric interview 6.0 (46)	Note 7	120	Yes/No	Kappa Cohen = 0.50–0.84
Personality disorders	Standardized Assessment of Personality Abbreviated Scale (47)	Note 8	8	Two point Likert-Scale	Cronbach's alpha = 0.68
Substance use disorders (SUDs)	Drug Abuse Screening Test-20 (48)	Note 9	20	Yes/No	Cronbach's alpha = 0.74
	Alcohol Use Disorders Identification Test (49)		10	2 or multiple choice questions	Cronbach's alpha = 0.88
Number of physical illnesses	CCHS (45)	Note 10	Yes/No		
Functional disability	WHO Disability Assessment Schedule 2.0 (50)	Note 11	12	Five-point Likert-scale 0 to 60 (where 0 = no disability; 60 = full disability)	Cronbach's alpha = 0.93–0.94
Suicidal ideation	CCHS (45)	Note 12		Yes/No	
**SERVICE USE VARIABLES**					
Frequency of service use	Service use Questionnaire: adapted from CCHS (51)	Note 13		Numerical	
Has a family physician	CCHS (45)	Note 14		Yes/No	

*Descriptive notes*:

*Note 1. Subjective quality of life consists of five domains: (I) daily life and social relations; (2) housing, neighborhood; (3) personal relationships; (4) spare-time activities; (5) autonomy*.

Note 2. Age was calculated from date of birth, as confirmed by participants.

Note 3. Sex as declared by participants.

Note 4. Criminal record as declared by participants.

Note 5. Homeless episodes as declared by participants.

Note 6.Housing type as declared by participants.

Note 7. M.I.N.I. is a short structured diagnostic interview, developed jointly by psychiatrists and clinicians in the United States and Europe, for DSM-IV and ICD-10 psychiatric disorders.

Note 8. The Standardized Assessment of Personality Abbreviated Scale was developed from the semi-structured interview Standardized Assessment of Personality.

Note 9. The Drug Abuse Screening Test-20 (DAST-20) is a screening tool. It is a 28-item self-report scale. Rating: 1–20; higher, greater drug use disorders.

The Alcohol Use Disorders Identification Test (AUDIT) Rating: 0–50; higher, greater level of substance use disorders.

Note 10. Number of physical illnesses as declared by participants.

Note 11. Short version: 12 items. It is used for all diseases, including mental, neurological, and addictive disorders. Scores assigned to each of the items—“none” (0), “mild” (1) “moderate” (2), “severe” (3), and “extreme” (4). 6 Domains of Functioning, include: cognition, mobility, self-care, getting along, life activities, and participation; Higher, less functional disability.

Note 12. Suicidal ideation as declared by participants.

Note 13. Frequency of service use as declared by participants.

*Note 14. Has a family physician as declared by participants*.

### Analyses

After cleaning the database for missing values and outliers, univariate analyses comprised of frequency distributions for categorical variables, and mean values with standard deviations for continuous variables were carried out, followed by Cluster verification analyses. Missing values (<5%) were randomly distributed and treated using the Expectation Maximization method. Clustering of participants was computed with the SPSS Statistics 24.0 package Two Step Cluster Analysis. QOL was the variable of interest. The choice of variables was based on their relevance to the homeless population according to the literature. Variables were organized as continuous or categorical variables. Categorical variables were entered in the program first, followed by continuous variables. The Log-likelihood method was used to determine inter-subject distance. Participant clusters were identified using Schwartz Bayesian criteria, with the final number of clusters set at four, according to their overall contributions to inter-Cluster homogeneity. An analysis of variance was also performed to test whether differences among profiles were statistically significant on QOL scores, followed by *post hoc* tests using the Bonferroni correction. Comparison analyses were run to assess statistical differences between clusters for each variable, using the Chi-square test for categorical variables, and ANOVA t-test for continuous variables.

## Results

The final sample consisted of 455 participants, of the 497 invited, for an overall response rate of 92%. Response rates for the four housing groups were: 94% (*n* = 229/243) for temporary housing; 90% (*n* = 140/156) for permanent housing with support; 79% (*n* = 41/52) for permanent housing without support; and 98% (*n* = 45/46) for homeless individuals using emergency shelters. Sample characteristics are presented in Table 2. Regarding sociodemographic variables, 60% of the sample were men, and 52% were 50 years of age, or older. Almost half had experienced a single homeless episode (46%), and 50% resided in temporary housing. In terms of clinical variables, 67% of participants reported personality disorders, 42% common MHDs (e.g., depression, anxiety), 39% SUDs, and 26% serious MHDs (e.g., schizophrenia, bipolar disorders). The mean for functional disability was 21, or moderate, on a 60-point scale. Concerning source of healthcare, 42% had a family physician. Participants had used community services an average of 72.5 times in the previous 12 months, public services 6.2 times, and the ER 1.9 times. Finally, the global mean score for QOL was 70.3/100.

**Table 2:** Participant characteristics.

		**Min**	**Max**	***n* (%)**	**Mean ± SD**
Quality of Life (QOL)		33.00	100.00		70.29 ± 10.00
**SOCIODEMOGRAPHIC VARIABLES**					
Age categories	18–39 years			28 (6.2%)	
	40–49 years			194 (42.6%)	
	50 and over			233 (51.2%)	
Sex	Women			181 (39.8%)	
	Men			274 (60.2%)	
Criminal record				101 (22.2%)	
Homeless episodes	1 episode			210 (46.2%)	
	2 episodes			79 (17.4%)	
	3–4 episodes			93 (20.4%)	
	5 episodes and over			73 (16%)	
Types of accommodation	Emergency shelter			45 (9.9%)	
	Temporary housing			229 (50.3%)	
	Permanent housing with support			140 (30.8%)	
	Permanent housing without support			41 (9.0%)	
**CLINICAL VARIABLES**					
Diagnoses	Common MHDs			190 (41.8%)	
	Serious MHDs			119 (26.2%)	
	Personality disorders			303 (66.6%)	
	SUDs			177 (38.9%)	
Number of physical illnesses		0.00	8.00		1.83 ± 1.63
Functional disability		11.00	49.00		20.55 ± 6.60
Suicidal ideation				101 (22.2%)	
**SERVICE USE VARIABLES**					
Source of health care:	Has a family physician			193 (42%)	
Frequency of service utilization	Public services	0.00	156.00		6.15 ± 13.87
	Community services	0.00	628.00		72.50 ± 115.43
	Emergency room	0.00	100.00		1.89 ± 6.84

Table 3 presents the four clusters identified in the sample based on QOL. Cluster 1 was composed of 128 participants (28% of total sample) with a mean overall score for QOL of 75.8 (SD = 10.7), ranking first of the four clusters. Cluster 2 consisted of 120 individuals (26% of total sample), had the lowest overall mean QOL score (mean = 66.0; SD = 11.0). Cluster 3 included 142 individuals (31% of sample) ranked second on QOL (mean = 70.4, SD = 3.1). Finally, Cluster 4 presented 65 participants (14% of total sample), ranked third on QOL (mean = 67.1; SD = 11.4). Analysis of variance comparing QOL scores among the four clusters revealed significant differences: F_(3, 454)_ = 26.54, *p* < 0.000. *Post hoc* tests using the Bonferroni correction revealed higher mean scores on QOL for Cluster 1 than for the other three Clusters (*p* < 0.000). Cluster 2 had lower scores than Cluster 3 (*p* < 0.001). Finally, the comparison between the mean scores for QOL for other Clusters (2 vs. 4, and 3 vs. 4) were non-significant (Tables 3, 4).

**Table 3:** Cluster analysis and comparison between clusters.

		**Cluster 1**			**Cluster 2**			**Cluster 3**			**Cluster 4**			**Combined**			**Total sample**
Quality of life (mean, SD)		75.84 ± 10.68 ^2, 3, 4^			65.99 ± 10.99 ^1, 3^			70.38 ± 3.12^1, 2^			67.12 ± 11.4^1^			70.29 ± 10			<0.00001
		n	% in row	% in column	n	% in row	% in column	n	% in row	% in column	n	% in row	% in column	n	% in row	% in column	
		128	28.10%		120	26.40%		142	31.20%		65	14.30%		455	100.00%		
**SOCIODEMOGRAPHIC VARIABLES**																	
Age categories	18–39 years	1	3.60%	0.78%	4	14.30%	3.33%	21	75.00%^1, 3, 4^	14.79%	2	7.10%	3.08%	28	100.00%	6.15%	<0.00001
	40–49 years	13	6.70%	10.16%	63	32.50%^1, 4^	52.50%	64	33.00%	45.07%	54	27.80%^1^	83.08%	194	100.00%	42.64%	
	50 years and over	114	48.90%^2, 3, 4^	89.06%	53	22.70%	44.17%	57	24.50%	40.14%	9	3.90%	13.85%	233	100.00%	51.21%	
Sex	Men	18	9.90%	14.06%	72	39.80%^1, 4^	60.00%	89	49.20%^4^	62.68%	2	1.10%	3.08%	181	100.00%	39.78%	<0.00001
	Women	110	40.10%^2, 3, 4^	85.94%	48	17.50%	40.00%	53	19.30%	37.32%	63	23.00%^1, 2^	96.92%	274	100.00%	60.22%	
Criminal record		9	8.90%^2, 3, 4^	7.03%	23	22.80%^1, 4^	19.17%	36	35.60%^1, 4^	25.35%	33	32.70%^1, 2, 3^	50.77%	101	100.00%	22.20%	<0.00001
Homeless episodes	1 episode	84	40.00%^2, 3, 4^	65.63%	36	17.10%	30.00%	61	29.00%	42.96%	29	13.80%	44.62%	210	100.00%	46.15%	<0.00001
	2 episodes	17	21.50%	13.28%	28	35.40%^1, 4^	23.33%	30	38.00%^1, 4^	21.13%	4	5.10%	6.15%	79	100.00%	17.36%	
	3–4 episodes	14	15.10%	10.94%	28	30.10%	23.33%	24	25.80%	16.90%	27	29.00%^1, 4^	41.54%	93	100.00%	20.44%	
	5 episodes and over	13	17.80%	10.16%	28	38.40%	23.33%	27	37.00%	19.01%	5	6.80%	7.69%	73	100.00%	16.04%	
Type of accommodation	Emergency shelter	16	35.60%	12.50%	7	15.60%	5.83%	12	26.70%	8.45%	10	22.20%	15.38%	45	100.00%	9.89%	<0.00001
	Temporary housing	41	17.90%	32.03%	49	21.40%	40.83%	88	38.40%^1, 2, 4^	61.97%	51	22.30%^1, 2, 3^	78.46%	229	100.00%	50.33%	
	Permanent Housing with support	61	43.60%^3, 4^	47.66%	51	36.40%^3, 4^	42.50%	25	17.90%	17.61%	3	2.10%	4.62%	140	100.00%	30.77%	
	Permanent Housing without support	10	24.40%	7.81%	13	31.70%	10.83%	17	41.50%	11.97%	1	2.40%	1.54%	41	100.00%	9.01%	
**CLINICAL VARIABLES**																	
Diagnoses	Common MHDs	35	18.40%^2, 3, 4^	27.34%	114	60.00%^1, 3, 4^	95.00%	6	3.20%^1, 2, 4^	4.23%	35	18.40%^1, 2, 3^	53.85%	190	100.00%	41.76%	<0.00001
	Serious MHDs	28	23.50%^2, 3^	21.88%	84	70.60%^1, 3, 4^	70.00%	0	0.00%^1, 2, 4^	0.00%	7	5.90%^1, 2, 3^	10.77%	119	100.00%	26.15%	
	Personality disorders	94	31.00%^2, 3, 4^	73.44%	112	37.00%^1, 3, 4^	93.33%	32	10.60%^1, 2, 4^	22.54%	65	21.50%^1, 2, 3^	100.00%	303	100.00%	66.59%	
	SUDs	53	29.90%^3, 4^	41.41%	62	35.00%^3, 4^	51.67%	6	3.40%^1, 2, 4^	4.23%	56	31.60%^1, 2, 3^	86.15%	177	100.00%	38.90%	
Suicidal ideation		14	13.90%^2, 3, 4^	10.94%	57	56.40%^1, 3^	47.50%	3	3.00%^1, 2, 4^	2.11%	27	26.70%^1, 3^	41.54%	101	100.00%	22.20%	<0.00001
Number of physical illnesses (Mean, SD)		2.22^3^	±1.62		2.23^3^	±1.99		1.24^1, 2^	±1.18		1.68^2^	±1.38		1.83	±1.63		<0.00001
Functional disability (Mean, SD)		17.71^2, 3, 4^	±5.3		25.27^1, 3, 4^	±8.15		19.21^1, 2^	±3.37		20.37^1, 2^	±6.53		20.55	±6.6		<0.00001
**SERVICE USE VARIABLES**																	
Source of health care: Has a family physician Frequency of service utilization (Mean, SD)		79	40.90%^3, 4^	61.72%	82	42.50%^3, 4^	61.72%	13	6.70%^1, 2, 4^	9.15%	19	9.80%^1, 2, 3^	29.23%	193	100.00%	42.42%	<0.00001
Public services		2.48^2, 3^	±4.28		8.2^1^	±12.39		6.7^1, 2^	±15.84		8.4	±21.19		6.15	±13.87		0.003
Community services		79.43	±125.1		74.36	±120.32		67.39	±112.65		66.58	±91.57		72.5	±115.43		0.816
Emergency room		0.88^2^	±1.53		2.07^1^	±3.64		2.81	±11.54		1.51	±2.16		1.89	±6.84		0.131

*χ^2^ Comparisons are done for each row reporting percentages for categorical variables and ANOVA t-test for continuous variables. Superscript indicates significant differences at p < 0.05 (look Table 4)*.

Cluster 1: “Mainly older women with one homeless episode, low functional disability and high QOL.”

Cluster 2: “Individuals with high functional disability, complex mental health problems and lowest QOL.”

Cluster 3: “Individuals living mainly in temporary housing with fewest SUDs and MHDs and moderate QOL.”

Cluster 4: “Mainly middle aged women living in temporary housing, with criminal records, personality disorders, SUDs, and low QOL.”

**Table 4:** Comparison tests between classes and variables.

		**Total sample**	**Class 1 vs*. 2***	**Class 1 vs. 3**	**Class 1 vs. 4**	**Class 2 vs. 3**	**Class 2 vs. 4**	**Class 3 vs. 4**
Quality of life		<0.0001^***^	<0.0001^***^	<0.0001^***^	<0.0001^***^	<0.0001^***^	0.987^***^	0.150^***^
Age categories		<0.0001^*^	<0.0001^*^	<0.0001^*^	<0.0001^*^	0.007^*^	<0.0001^*^	<0.0001^*^
Sex		<0.0001^*^	<0.0001^*^	<0.0001^*^	0.022*^*^	0.657^*^	<0.0001^**^	<0.0001^**^
Criminal record		<0.0001^*^	0.004^*^	<0.0001^*^	<0.0001^*^	0.232^*^	<0.0001^*^	<0.0001^*^
Homeless episodes		<0.0001^*^	<0.0001^*^	0.003^*^	<0.0001^*^	0.170^*^	<0.0001^*^	<0.0001^*^
Housing		<0.0001^*^	0.153^*^	<0.0001^*^	<0.0001^*^	<0.0001^*^	<0.0001^*^	0.002^*^
Mental health disorders (MHDs)	Common MHDs	<0.0001^*^	<0.0001^*^	<0.0001^*^	<0.0001^*^	<0.0001^*^	<0.0001^*^	<0.0001^*^
	Serious MHDs	<0.0001^*^	<0.0001^*^	<0.0001^**^	0.058^*^	<0.0001^**^	<0.0001^*^	<0.0001^**^
	Personality disorders	<0.0001^*^	<0.0001^*^	<0.0001^*^	<0.0001^*^	<0.0001^*^	0.052^**^	<0.0001^**^
	SUDs	<0.0001^*^	0.105^*^	<0.0001^*^	<0.0001^*^	<0.0001^*^	<0.0001^*^	<0.0001^*^
Suicidal ideation		<0.0001^*^	<0.0001^*^	0.004^**^	<0.0001^*^	<0.0001^**^	0.437^*^	<0.0001^**^
Number of physical illnesses		<0.0001^***^	1.000^***^	<0.0001^***^	0.095^***^	<0.0001^***^	0.206^***^	0.164^***^
Functional disability		<0.0001^***^	<0.0001^***^	0.038^***^	0.031^***^	<0.0001^***^	<0.0001^***^	0.698^***^
Has a family physician		<0.0001^*^	0.275^*^	<0.0001^*^	<0.0001^*^	<0.0001^*^	<0.0001^*^	<0.0001^*^
Frequency of utilization	Public services	0.003^***^	<0.0001^***^	0.015^***^	0.162^***^	0.948^***^	1.000^***^	0.993^***^
	Community services	0.816^***^	1.000^***^	0.957^***^	0.961^***^	0.997^***^	0.997^***^	1.000^***^
	Emergency room	0.131^***^	0.007^***^	0.268^***^	0.217^***^	0.978^***^	0.722^***^	0.732^***^

* *Pearson test*;

** *Fisher test*;

*** *ANOVA t-test*.

Tests confirming the cluster analysis indicated that socio-demographic variables (age, sex, criminal record, homeless episodes, type of accommodation), clinical variables (suicidal ideation, number of physical illnesses, and functional disability), and frequency of services use (public services) differed significantly across clusters (Table 3). Cluster 1 included predominantly women 50 years old and over, who had experienced one episode of homelessness. Cluster 1 also had the lowest proportion of individuals with criminal records and the lowest functional disability scores. As compared with those in Clusters 3 and 4, more Cluster 1 participants resided in permanent housing with support; they reported a greater number of physical illnesses, but more often had a family physician. Compared with Clusters 2 and 3, Cluster 1 participants also made less use of public services. Finally, compared with Cluster 2, Cluster 1 participants reported lower ER use. Cluster 1 was labeled: “*Mainly older women with one homeless episode, low functional disability and high QOL*”

Cluster 2 had the highest proportions of individuals with MHDs (both common and serious MDs) and suicidal ideation, as well as higher functional disability scores than individuals in the other three clusters. As opposed to both Clusters 1 and 4, Cluster 2 had a higher proportion of individuals with two episodes of homeless, and more with five homeless episodes and over. Compared to Clusters 3 and 4, Cluster 2 also had a higher proportion of individuals with a family physician, and more residing in permanent housing with support. Finally, compared with Cluster 1, Cluster 2 individuals used public services with greater frequency. Cluster 2 was labeled: “*Individuals with higher functional disability, complex mental health problems and lowest QOL*.”

Cluster 3 mainly consisted of individuals residing in temporary housing who had the lowest proportion of SUDs, MHDs (both common and serious MDs), personality disorders, and suicidal ideation among the 4 clusters. As compared with Clusters 1, Cluster 3 had a higher proportion of individuals with a criminal record and with two homeless episodes. Individuals from Cluster 3 were also less affected by physical illnesses. Fewer had a family physician compared with individuals in Clusters 1 and 2. Cluster 3 was labeled: “*Individuals living mainly in temporary housing with fewest SUDs and MHDs and moderate QOL*.”

Finally, Cluster 4 had a higher proportion of women between 40 and 49 years of age, who had experienced one homeless episode. More had a criminal record, resided in temporary housing, and had personality disorders and SUDs as compared with individuals in the other three clusters. Cluster 4 was labeled: “*Mainly middle aged women living in temporary housing, with criminal records, personality disorders, SUDs, and low QOL*.”

## Discussion

This study developed a typology for a sample of homeless individuals on the basis of QOL in relation to sociodemographic, clinical, and service use characteristics. Four clusters were identified, each with distinct features. Mean QOL scores varied from 66.0 to 75.8 (*M* = 70.3), which was lower than QOL scores for the general population in a Quebec epidemiological area, at 78 (52).

Our results showed marked differences among the four clusters in terms of sex, age, presence or absence of a criminal record, episodes of homelessness, and residence in temporary vs. permanent housing, number of physical illnesses, MHDs, or SUDs, as well as sources of health care and frequency of public service use in the previous year.

Cluster 1 differed from other clusters in terms of mean QOL, but also on sociodemographic and clinical characteristics. Cluster 1 mainly included older women who had experienced one episode of homelessness; they had the lowest scores on disability and criminal record as well the highest QOL. These results seem to confirm that high QOL among homeless individuals was associated with older age and female gender, as identified in previous research (53). Moreover, this cluster with the higher QOL also included those less affected by functional disability, which suggests that functional disability negatively influenced QOL (22).

Cluster 1 showed very marked differences from Cluster 2, which had the lowest QOL in relation to socio-demographics (sex, age, criminal record number of homeless episodes), and clinical variables (both common and serious MHDs, personality disorders, suicidal ideation, and functional disability) and on the frequency of public service use. Cluster 2 mainly consisted of individuals with a high prevalence of MHDs (common, serious, personality disorders), and functional disability. MHDs have been associated with lower QOL (14, 17). Previous studies also found that the presence of MHDs was associated with increased access to public health services; including primary care (54). The presence of multiple MHDs among homeless individuals also increases the risk of suicide (24). As well, functional disability, higher in Cluster 2, is a frequent result of both medical and psychiatric conditions, creating barriers to employment, and perpetuating the cycle of homelessness (55). A US study estimated that 37% of homeless individuals have a functional disability as compared with 25% of individuals living in poverty, and 15% of the general population (56). The fact that Cluster 2 individuals resided mainly in permanent supported housing, as in Cluster 1, seemed to indicate that QOL was not automatically associated with type of accommodation in homelessness. Similarly, QOL was not automatically associated with a regular source of health care, as the two clusters with higher (Cluster 1) vs. lower (Cluster 2) QOL reported similar proportions of individuals with a family physician.

Cluster 3 was second in terms of QOL, with moderate scores. Cluster 3 was mainly composed of individuals with few MHDs and SUDs who residing in temporary housing. The higher QOL than reported in Cluster 3 may have been due to the very low numbers of MHDs, SUDs, physical illnesses and suicidal ideation in this cluster. Individuals with MHDs or SUDs tend to report low QOL scores (13, 17, 21, 22). The low prevalence of MHDs and SUDs may also explain the low use of healthcare resources, such as family physicians and public services, in this cluster.

QOL scores for Clusters 3 and 4 showed no significant differences. These Clusters were similar in terms of the number of physical illnesses, levels of functional disability, and frequency of service utilization. However, major differences emerged on other variables between Clusters 3 and 4, the later consisting mainly of women greatly affected by personality disorders, SUDs, common and serious MHDs and suicidal ideation. The prevalence of common MHDs, and personality disorders was higher in women than men (57). Among women, MHDs, SUDs as well as violence are main causes of homeless (58). A systematic review of the literature on homeless women veterans also revealed that women were more likely to be affected by MHDs than men (59), and were more often involved in the justice system, in addition to having relatively higher rates of MHDs and SUDs (57).

Clusters 2 and 4 accounted for the lowest QOL scores, with no significant differences between them. These clusters were similarly and strongly characterized by high rates of personality disorders, SUDs, common MHDs, suicidal ideation, and high functional disability among their respective constituents. MHDs and SUDs have been identified in association with poor QOL, with a correspondingly high negative impact on family relationships and on employment status (17, 60, 61). Individuals in the two clusters also had similarly high rates of public services use, which suggests that frequency of service use was more related to clinical variables than socio-demographic variables or type of housing.

Finally, compared with Cluster 1, Cluster 4 consisted almost exclusively of middle aged women living in temporary housing, who had low QOL scores. As well, all Cluster 4 participants had a disproportionately high prevalence of personality disorders and SUDs, which is interesting as individuals with dual diagnoses are known to be high service users (62). Cluster 4 also had a disproportionate number of individuals with SUDs and criminal records. These results were similar to results of other studies underlining that SUDs increased vulnerability among homeless women, making them more prone to participate in drug-related crimes (63, 64).

## Strengths and Limitations

This first study to profile QOL in a homeless sample included individuals living in different types of accommodation (emergency shelter, temporary housing, and permanent housing with and without support). In addition, the participation rate (92%) in this study was very high; only 42 of the 497 participants invited to the study refused to participate. Moreover, this study provided highly relevant insights into different aspects of homelessness and their associations with QOL, identifying sociodemographic, clinical, and service use characteristics affecting QOL in homelessness.

This study had also limitations that should be noted. The main limitation concerned the modest number of variables that could be introduced into the cluster analysis. Second, due to the convenience sampling, our results may not be generalizable. Third, our results emanating from data collected in Quebec may not be generalizable to other jurisdictions. Fourth, the study used cross-sectional and self-reported data. A longitudinal study could have better highlighted the causal relationships between QOL and the selected independent variables. Fifth, some housing groups, such as emergency shelters, were less represented than others in this study. Finally, while the sample was more evenly distributed in terms of sex, there were relatively fewer young people than those in older age categories.

## Conclusion

The use of cluster analysis provides insight into the differences among homeless individuals in terms of QOL, taking into account sociodemographic, clinical, and service use variables. A better understanding of QOL in different homeless groups may help inform policy and service planning, while better responding to the need for client-focused healthcare that is sensitive to group differences. Our results suggest that older age may have a positive influence on QOL in women, while clinical characteristics, such as MHDs, SUDs, and high functional disability scores may influence QOL negatively, as the two clusters where individuals were more affected by complex mental health problems revealed the lowest QOL scores. Moreover, type of accommodation, having a family physician, and frequency of service utilization seemed not to have a direct impact on QOL. Temporary and permanent housing may both positively influence QOL but only among individuals without complex health problems.

Our findings reinforce the importance of disseminating specific programs adapted to the diverse profiles within homeless populations, with a view toward increasing their QOL. For Cluster 1, use of a family physician may be sufficient to meet the needs of that fairly functional clientele; whereas in Cluster 3, strategies such as the deployment of outreach workers may be needed to encourage service use. Programs that promote social integration may influence QOL in Cluster 4, as this group included a high proportion of women with criminal records and SUDs. Finally, assertive community treatment should be considered as an effective strategy for Cluster 2 individuals affected by both multiple MHDs and high functional disability.

## Author Contributions

LG, GG, and M-JF: study design and analyses, interpretation of data, and preparation of manuscript. J-MB: data analysis. HD: revision of manuscript.

### Conflict of Interest Statement

The authors declare that the research was conducted in the absence of any commercial or financial relationships that could be construed as a potential conflict of interest.
